# Implication of CDKN2A in cuproptosis through defining cuproptosis-related gene signature in ovarian cancer

**DOI:** 10.7150/jca.115374

**Published:** 2025-07-24

**Authors:** Beilei Zhang, Zhaojie Yang, Yinuo Zheng, Yongchao He, Yulu Yan, Jiarui Song, Fu Wang, Ruifang An

**Affiliations:** 1 Department of Obstetrics and Gynecology, The First Affiliated Hospital of Xi'an Jiaotong University, Xi'an 710061, Shaanxi, China.; 2 Institute of Medical Engineering, School of Basic Medical Sciences, Xi'an Jiaotong University, Xi'an 710061, China.

**Keywords:** Ovarian cancer, Cuproptosis, Gene signature, CDKN2A

## Abstract

Cuproptosis is a kind of programmed cell death in which copper reacts with the cycloaliphatic component of the tricarboxylic acid (TCA) cycle. In this study, we devised a predictive model and a theoretical framework to examine the variations in the expression of the cuproptosis-related genes (CRGs) in ovarian cancer. Through screening the 11 CRGs, all samples were segmented into two risk groups and a prognostic model was built. Among the 11 CRGs, 10 genes showed a significant relationship with survival probability, demonstrating the model had good prediction ability and high accuracy. Age and FIGO stage were discovered to be strongly correlated with patient survival time by means of univariate Cox regression analysis. The patients over 65 in FIGO stages IIIA-IV had an increased risk. The enrichment analysis showed that the main metabolic pathways were those related to drug metabolism, tissue development, tyrosine metabolism and retinol metabolism. The PPI networks revealed that CDKN2A was the key gene. Finally, the *in vitro* and *in vivo* functional assays demonstrated that cuproptosis induced by CuET agent treatment could significantly inhibit ovarian cancer cell viability, migration and invasion as well as xenografted tumor growth where the CDKN2A expression level increased. Our results indicate that the comprehensive definition of differentially expressed CRGs in ovarian cancer will provide new insights for clinical remedy of ovarian cancer.

## Introduction

Copper is a fundamental element for our body 1. The concentration of copper in normal cells is terribly low, and its primary mechanism is to prevent detrimental accumulation of free copper in cells with dynamic balance across concentration gradients, thereby maintaining the copper homeostasis of cells 2, 3. A novel cell death mechanism, cuproptosis, has been lately confirmed 4. Different from apoptosis, ferroptosis and pyroptosis 5, can contribute to protein toxicity stress by directly binding to lipoylated proteins, a key enzyme in the TCA cycle, and can moreover induce mitochondrial metabolic dysfunction. Moreover, through the production of reactive oxygen species (ROS), cuproptosis can also induce tumor cell death via activating apoptosis signaling pathways, which makes it a novel mechanism for anti-tumor study 6.

Ovarian cancer (OV) is a malignant ovarian tumor, of which 90%-95% is primary OV, and the remaining 5%-10% is primary cancer metastases in the ovarian system 7. OV's early diagnosis is laborious because of lacking symptoms and hidden onset in the early stage, as well as the lack of effective early diagnosis methods. At the time of the initial diagnosis, there are 60%-70% of patients already in the advanced stage, which makes the healing efficacy poor 8-10. Most patients experience disease relapse within 2 years, and there is a lack of an effective therapeutic schedule for recurrent OV 11. Therefore, although the incidence rate of cervical cancer and endometrial cancer is higher than that of OV, OV still has a higher mortality rate than the sum of the first two, making it the most common cause of death among various gynecological tumors 12-14.

OV is pernicious, which can't be accurately and effectively diagnosed in its early stages and has a poor prognosis 15-18. Copper is an essential element in the body and is critical in many biological processes 19, 20. Cuproptosis, a new form of programmed cell death has been confirmed 4. However, the importance of copper in the induction of cell death-related gene expression in OV has not been assessed. With the increasing popularity of DNA sequencing, high-throughput proteomics and metabolomics, the key prognostic genes of cuproptosis-related OV can be found by high-throughput gene chip technology and bioinformatics, and the prognostic model of CRGs in OV can be constructed 21, which provides novel ideas for the clinical diagnosis and treatment of OV.

There have been some reports of copper death in OV. Kang et al. found that the liposome DQ/CuClz complex induced cell death through ROS mediated REDOX homeostasis in cancer cells, showing promising anticancer potential 22. Kordes tani et al. confirmed that Cu(Ⅱ) complex 1 induced stronger apoptotic reaction, increased mitochondrial membrane depolarization, and increased intracellular ROS levels, thus leading to the death of A2780 OV cells 23. Zhang et al. established a risk prediction model for OV based on 13 CRGs, including APT7A, LISA, DLAT, SLC31A1, FDX1, DLD, ATP7B, and PDHB, and based on their expression differences in OV tissues, a risk scoring model was constructed 24. The model can predict the effectiveness of chemotherapy in OV patients and guide drug selection. Research on OV cells using these genes-based copper death regulators shows that certain copper complexes [Cu(Ⅱ) complexes] can inhibit OV cell proliferation with lower toxicity than non-essential metals such as platinum, suggesting that targeted copper death therapy for OV has certain prospects.

In this study, the differential expressions of CRGs in OV were analyzed comprehensively, as well as 62 CRGs were screened progressively employing univariate Cox regression analysis and LASSO regression analysis. Accordingly, we constructed the risk model, and Kaplan-Meier curves and receiver operating characteristic (ROC) curves at 1, 3 and 5 years were plotted for analysis. The results indicated that CRGs have potential value and effect on OV. It is of certain authenticity and feasibility that these CRGs can be utilized to construct the prognosis model. Eleven CRGs were identified by multivariate Cox regression analysis which can be used for constructing the prognostic model in OV. Through GO and KEGG pathway enrichment analysis of differentially expressive CRGs in OV, we found that these genes were principally enriched in tissue development, oxidoreductase activity, drug metabolism, tyrosine metabolism and retinol metabolism. The above metabolic processes and enzyme activities have an inevitable correlation with the occurrence and development of OV. In addition, through the construction of protein-protein interaction (PPI) networks, the core gene CDKN2A was identified, which plays a significant part in biological signaling, regulation of gene expression, metabolism of energy and substances, and regulation of the cell cycle.

## Materials and Methods

### Data acquisition and preprocessing

We initially downloaded the RNA-seq data of 429 OV samples along with their corresponding clinical data from TCGA database (https://www.genome.gov/) and extracted the FPKM 25 as our testing groups. Subsequently, we employed the ID conversion using the "rjson" package and successfully procured the clinical data matrix and gene expression matrix. We also downloaded the mRNA chip data of serous OV and normal ovarian cells with ID numbers of GSE14407, GSE18520 and GSE54388 from GEO database(http://www.ncbi.nlm.nih.gov/geo/) 26.

In order to prevent data from overfitting, we eliminated the duplicated and missing data, standardized the expression matrix and filled in the missing values utilizing "impute" package and set the "Number of neighbors" to 15 to supplement the data. The standardization method was as follows: after adding 1 to each data, log2(X+1) was taken with base 2, where X was the expression data of different genes in different samples. CRGs were found by searching the Genecards database(https://www.genecards.org) with the term "copper induced cell death". Utilizing Excel's "VLOOKUP" and "HLOOKUP" functions, we matched and combined the gene expression matrix with the clinical data matrix, and filtered the data of CRGs. The resultant matrix served as the basis for further study.

### Construction and identification of the prognosis model

To evaluating the relevance between the expression of these CRGs and OV patients' overall survival, we employed the packages "survival" and "survminer" in the R Programming Language to execute a univariate Cox regression analysis 27 on CRGs, and the genes with p < 0.05 were screened out.

The LASSO regression analysis study was carried out using the "survival", "glmn" and "pacman" packages, with the intention to further filter the CRGs, build a LASSO regression model and determine the risk values of each gene 28, 29. Each OV patient's risk score was determined grounded on the results of the LASSO regression analysis. Risk score 30 of each OV sample was calculated using the following formula: Risk score = regression coefficient for each cuproptosis-related gene × each sample's expression level for each predictive gene. In accordance with the intermediate values of the risk scores, two groups of OV patient samples were generated, one representing high risk and the other low risk. After that, the Kaplan-Meier curve and ROC curve, which could be applied to assess the prognosis and forecasting capacity of the LASSO regression model for CRGs were drawn subsequently using the "survminer" package and the "timeROC" package.

Afterwards, we examined these genes using multivariate Cox regression analysis. Utilizing the "survival" as well as "survfit" functional packages, taking into account the conditions of survival time, survival status along with gene data, we executed a multivariate Cox regression analysis with the aim of assessing the predicting effectiveness of these characteristics in OV patient samples. We defined p<0.05 as the screening criterion, CRGs meeting the criteria were considered as the optimum prognostic genes, and then we drew the forest map using R package "ggplot2" for them. The Kaplan-Meier curves 31 of each optimum prognostic CRGs were plotted to observe their prognostic ability for OV. After establishing the connection between the expression of each gene and sample's overall survival, we determined the risk score for these CRGs. As a consequence, these OV samples were split into high- and low-risk categories on the grounds of the intermediate value of the risk scores. Accordingly, the Kaplan-Meier curve was plotted. Moreover, we drew ROC curves 32 so as to calculate the prognostic accuracy of these CRGs. Afterwards, we analyzed the relationship between patients' risk scores, distribution of survival time, and gene expression to further verify the prognostic model.

### Establishment of a nomogram model

To pinpoint independent predictors of prognosis and to confirm the independent prognostic significance of the gene signature, both univariate and multivariate Cox regression analyses were conducted within the TCGA dataset. These analyses encompassed the prognostic gene signature along with various clinicopathological factors, such as age, FIGO stage, tumor grade, tumor size, histological subtype, chemotherapy history, radiation therapy history, targeted molecular therapy history, tobacco smoking history, and alcohol consumption history. A P-value threshold of less than 0.01 was set to determine statistical significance. Variables that achieved P < 0.01 in the univariate analysis were subsequently incorporated into the multivariate Cox regression analysis. Following the multivariate analysis, all independent prognostic factors and pertinent clinical variables were utilized to develop a prognostic nomogram. This nomogram was constructed using a stepwise Cox regression model in R software to forecast the 1-, 3-, and 5-year overall survival rates for OV cancer patients in the TCGA dataset 33, 34. The predictive accuracy of the nomogram was assessed through calibration curves and clinical decision curves, which effectively demonstrated the model's discriminatory power, calibration accuracy, and clinical applicability 35.

### Analysis of the differential expression and functional enrichment

For the sake of screening out the differential expressed genes on OV, we imported the chip data of GSE14407, GSE1852 and GSE5438 into R software, where the "affy" package is used to normalize the original data of the above datasets, and then we applied "Limma" package to manage the difference of GEO microarray data. The filtering threshold was confined to p < 0.01 after calibration, and the differential expression multiple | logFC | ≥ 4. Besides, the volcanic plots of the differentially expressive genes in each dataset were drawn through the "ggplot 2" package. A heatmap of the up or down-regulated expression genes was plotted using the "pheatmap" package. In addition, we intersected the up- or down-regulated expression genes in the four datasets above using the "venndigram" package to get the common differentially expressive genes, and finally got the common differentially expressive genes in OV associated with cuproptosis.

To gain insight into the biological functions and pathways of CRGs, we performed functional enrichment analyses 36 of these CRGs in OV, including the Gene Ontology (GO) 37, 38 and Kyoto Encyclopedia of Genes and Genome (KEGG) 39, 40. GO assays are typically used to test the enrichment of gene ontology entries in gene collections and predict common features of genes in biological processes (BP), molecular functions (MF), and cell components (CC), while KEGG is used to analyze the role of genes in metabolism and signaling pathways.

To be specific, employing the functional packages "org.Hs.eg.db" and "clusterProfiler" in the R Programming Language, we performed GO enrichment analysis, which has three ontologies that respectively describe the molecular function (MF), cellular component (CC), and involved biological process (BP) of a gene 41. After that, we successfully derived the latest information of KEGG Pathway gene annotation from the KEGG REST API (https://www.kegg.jp/kegg/rest/keggapi.html). Additionally, the "clusterProfiler" 42 package was applied to carry out the enrichment analysis. When a gene met the filtering criteria of p < 0.05 and FDR < 0.1, it was deemed statistically significant, and the outcomes of the enrichment were presented on bubble charts.

### Construction of protein-protein interaction (PPI) network

STRING database 43 (STRING: functional protein association networks (https://string-db.org/)) was utilized to establish a PPI network 44 of these differentially expressed genes in OV samples, and the visualization was achieved by Cytoscape software (version 3.4.0, http://chianti.ucsd.edu/cytoscape-3.4.0/). In addition, we applied the CytoNCA plug-in (Version 2.1.6, http://apps.cytoscape.org/apps/cytonca) to analyze the nodal topological properties of the network, where Degree was used as the main attribute. CytoNCA is a cytoscape plugin for centralized analysis and evaluation of biological networks, so that the key nodes could be filtered from the interworking networks. The larger value of the attribute proved the greater role of the gene in the network. The top 67 genes were selected and the key differentially expressed CRGs were obtained.

### Cell lines and reagents

The human OV cell line ES-2 and cervical cell line HUCEC (Human Uterine Cervical Epithelial Cells) were maintained in our lab. ES-2 cells were incubated in McCoy's 5A Medium (Gibco, USA) supplemented with penicillin/streptomycin antibiotics (100 U/mL penicillin, 100 g/mL streptomycin, Gibco, USA) and 10% fetal bovine serum (HyClone) in a humidified 5% CO_2_ incubator at 37 °C. HUCEC cell were incubated in F-12K Medium adding 0.1 mg/mL Heparin Solution, 500 μL of 30 mg/mL Endothelial Cell Growth Supplement (ECGS) and 10% fetal bovine serum (HyClone) in a humidified 5% CO_2_ incubator at 37°C. Cell copper Content Assay Kit (BC5750) was purchased from Solarbio (Beijing, China). CDKN2A/p16INK4a Antibody (AF5484) were acquired from affinity (Cincinnati, USA). anti-Beta Actin (20536-1-AP) antibodies were obtained from Proteintech Group (Wuhan, China). pCMV3-CDKN2A-C-Flag (HG29840-CF) plasmid was purchased from SinoBiological (Beijing, China). BCA protein assay kit (23227) was purchased from Thermo Scientific (New York, USA). Tween 80 (T8360), Polyethylene glycol 300 (IP9020), and Kanamycin Sulfate (IK0030) were obtained from Solarbio (Beijing, China). HRP-conjugated secondary antibody (10702-MM01E) was purchased from Santa Cruz (New Jersey, USA). Western Bright ECL reagent (K-12045-D20) was purchased from Advansta (California, USA). Protease Inhibitor Cocktail (HY-K0010) was purchased from MedChemExpress (New Jersey, USA).

### MTT assay

ES-2 cells were plated in 96-well plates at a density of 1 × 10⁴ cells per well. On the following day, the culture medium was refreshed with medium supplemented with varying concentrations of Copper (II) Diethyldithiocarbamate (CuET). Following a 12 h incubation period, the cells were subsequently treated with 100 mL of medium containing 0.5 mg/mL MTT for an additional 4 h. Afterward, the supernatant was discarded from each well, and the formazan crystals produced by viable cells were dissolved in 100 µL of DMSO. The absorbance at 490 nm was then measured using a microplate reader (SuPerMax 3100, Flash, China).

### Reverse transcription and quantitative PCR (qPCR)

To detect the mRNA expression level of CDKN2A, the total RNA obtained from ES-2 cells treated with CuET (0.5 μg/mL) was extracted using Trizol reagent (Invitrogen, GrandIsland, NY) according to the manufacturer's instructions. 2 μg of total RNA was reverse-transcribed into cDNA using a cDNA synthesis kit (SIMGEN, China). After that qPCR was performed with PCR Mix (Biosharp, China) and CDKN2A primers (Beyotime, China) at 95 °C for 5 min, 40 cycles of 95 °C 30 s, 60 °C 30 s using Quant Gene 9600 PCR system (Bioer, China). GAPDH served as the endogenous control for normalization. The primer sequences are as follows: CDKN2A Forward Sequence: CTC GTG CTG ATG CTA CTG AGG A; Reverse Sequence: GGT CGG CGC AGT TGG GCT CC; GAPDH Forward Sequence: GTC TCC TCT GAC TTC AAC AGC G, and Reverse Sequence: ACC ACC CTG TTG CTG TAG CCA A.

### Wound healing assay

ES-2 cells were plated in 12-well plates and cultured overnight to attain nearly 100% confluence. A scratch was made across the cell monolayer using a p10 pipette tip, and each well was washed twice with PBS to remove dislodged cells. The cells were subsequently cultured in DMEM medium supplemented with 1% FBS and CuET (0.5 μg/mL). Wound closure was monitored microscopically at 0 and 12 hours, and the relative wound healing area was quantified using ImageJ software.

### Cell migration assay

A 24-well plate with 8.0 μm membrane Transwell chamber was prepared. Cells were starved (without serum medium) for 24h for cell migration experiment, and 5×10^4^ cells /200 μL were inoculated in the Transwell chamber. 600μL medium containing 10% fetal bovine serum was added into the lower room, cultured in an incubator for 48 h, then removed, fixed with 4% paraformaldehyde, stained with 0.5% crystal violet, observed and photographed under a microscope.

### Western-blotting analysis

To prepare whole cell lysates, cells were subjected to RIPA buffer containing a protease inhibitor (MCE). Following this, the cellular extracts underwent centrifugation at 13,000g for 15 min at 4 ℃. The protein concentration in the resulting supernatant was measured using a BCA protein assay kit (PIERCE). Subsequently, 10 mg of the cell lysates were heated at boiling temperature for 10 min and resolved by SDS-PAGE. The proteins were then transferred to a PVDF membrane. Western blotting was performed with primary antibodies specific to CDKN2A and β-actin. The membrane was further incubated with an HRP-conjugated secondary antibody (Santa Cruz) for 1 h. After rinsing with PBST, the protein bands were revealed using the Western Bright ECL reagent (Advansta) and captured using a ChemiDoc Touch Imaging System (Bio-Rad).

### Xenograft mice tumor model

The animal studies were carried out and authorized by the Biomedical Ethics Committee of the Health Science Center at Xi'an Jiaotong University. Six-week-old female Balb/c-nu mice were sourced from Changsheng Biotechnology and housed under a regulated 12-hour light/dark cycle, with unrestricted access to food and water. Each mouse received an intracranial injection of 5×10^6^ ES-2 cells. A total of eight mice were allocated to each experimental group. Once the average tumor volume reached 100 mm³, the mice were randomly assigned to three distinct groups. The control group received 0.9% saline, the second group was administered CuET at a dose of 5 mg/kg, and the third group received CuET at a dose of 15 mg/kg. After a period of four weeks, the mice were humanely sacrificed, and their tumors were harvested for further analysis.

### Statistical analyses

For this investigation, statistical evaluations were performed utilizing SPSS version 23.0, where quantitative information was presented as mean ± SD. Notable disparities were determined through either a one-way ANOVA or an unpaired Student's t-test. The significance thresholds were set at: *p < 0.05, **p < 0.01, and ***p < 0.001 for each experiment.

## Results

### Construction of prognosis and multivariate cox regression models

We downloaded the clinical data and the data on gene expression in OV samples from TCGA database. After conducting data extraction and ID conversion using the "rjson" package, we obtained gene expression matrix with data of 429 samples. Then we prevent data overfitting by eliminating the duplicated and missing data, standardizing the expression matrix and filling in the missing values. Consequently, we acquired the processed expression matrix including 425 samples. Furthermore, Sangerbox platform 45 was used to standardize the data of expression matrix. 2029 CRGs were retrieved from Genecards database, and we matched and combined these CRGs with their related expression and clinical data. Subsequently, 1930 CRGs and their relevant data were acquired. A univariate Cox regression analysis was implemented on the CRGs for the purpose of further filtering these genes with great prognostic value 46. According to the analysis, 200 CRGs genes were indicated to have a moderately obvious correlation with patients' overall survival (OS) for OV (p < 0.05). The genes along with their related data were exhibited in **Table S1**.

**Figure 1:**
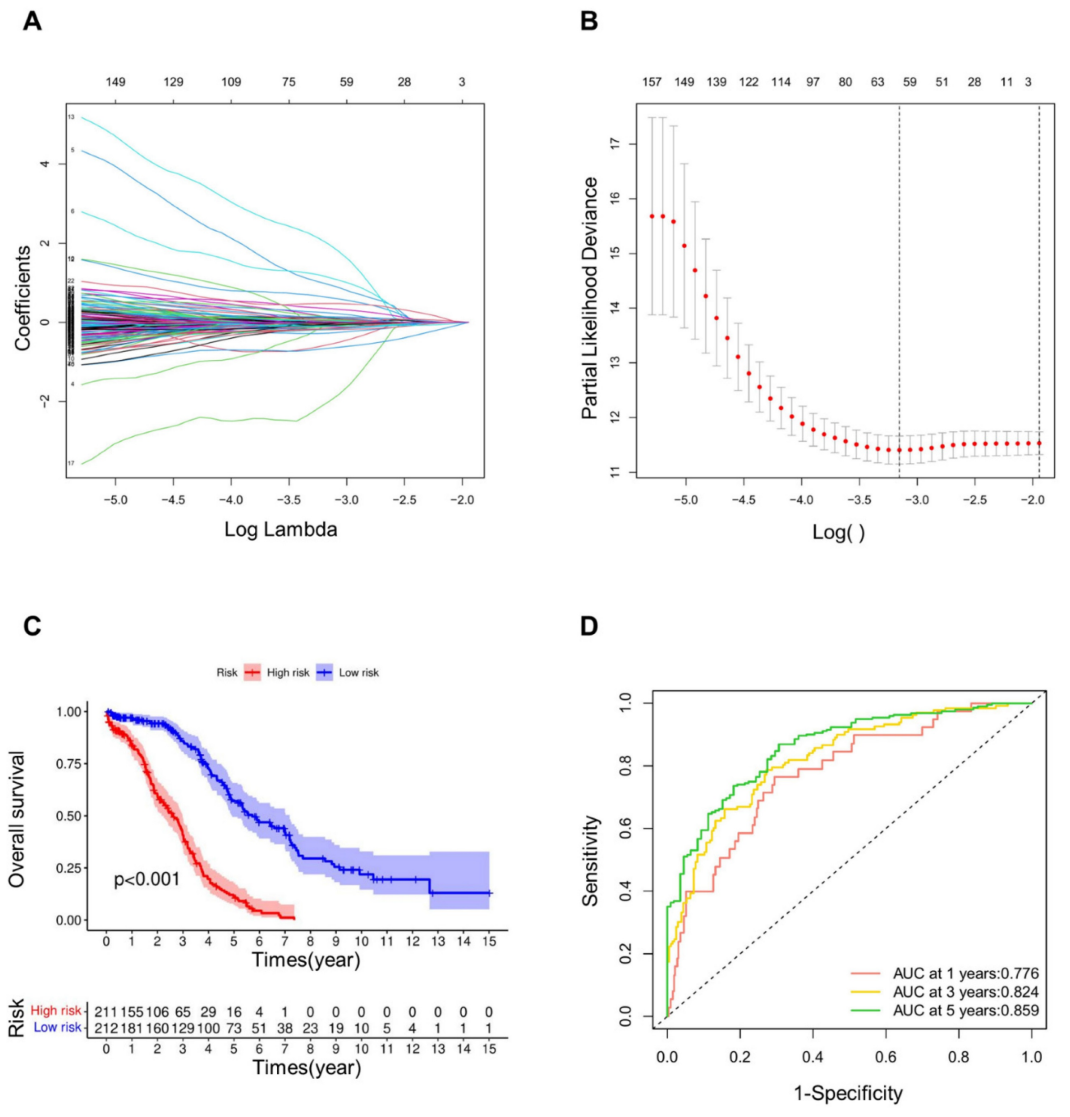
** Construction and verification of prognostic signature. (A)** The LASSO coefficient diagram of cuproptosis-related genes in OV. **(B)** Misclassification error against log(λ) is plotted. **(C)** Kaplan-Meier curves of high and low risk groups in LASSO model. **(D)** ROC curves of OV patients in LASSO model at 1, 3 and 5 years.

We further screened CRGs using LASSO regression analysis (**Fig. 1A, B**), thus obtaining 62 CRGs (**Table S2**). The risk score of each sample was determined on the ground of the built risk model. As an outcome, the samples were consequently separated into high and low groups. According to the Kaplan-Meier curves (**Fig. 1C**), the low-risk group's overall survival rate was substantially greater than that of the high-risk group at the significance of p < 0.001. ROC curve (**Fig. 1D**) demonstrated the AUC values, which presented the area under the ROC curve. The AUC values at years 1, 3, and 5 were 0.776, 0.824 and 0.859, respectively. Since all of these values were greater than 0.75, it could be demonstrated that the precision and dependability of this signature for patient prognosis was reliable.

**Figure 2:**
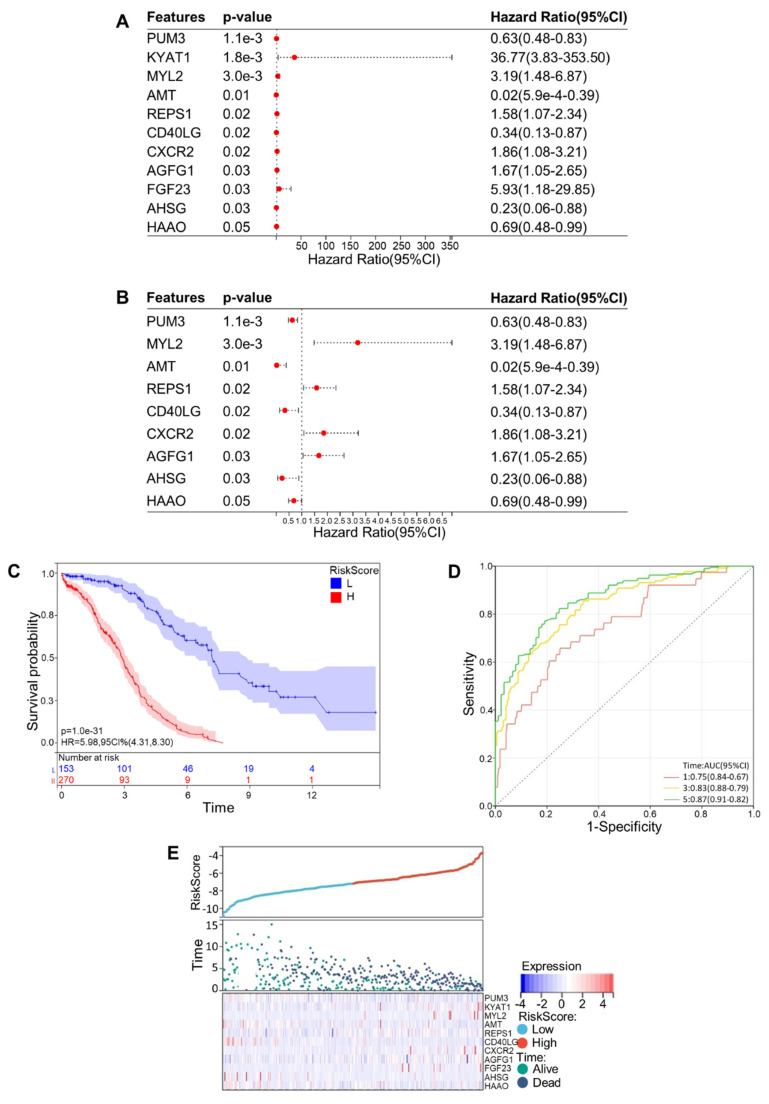
** Construction and verification of multivariate cox regression model. (A)**Forest map of 11 cuproptosis-related genes obtained from multivariate Cox regression analysis. **(B)** In order to make the forest map obtained by multivariate Cox regression analysis more obvious, two genes with excessive confidence interval were deleted from Figure 2(a). **(C)** Kaplan-Meier curves of high and low risk groups in multivariate Cox regression model. **(D)** ROC curves of OV patients in multivariate Cox model at 1 year, 3 years and 5 years. **(E)** Risk scores, survival time distribution and expression of high-risk and low-risk groups in the multivariate Cox model.

**Figure 3:**
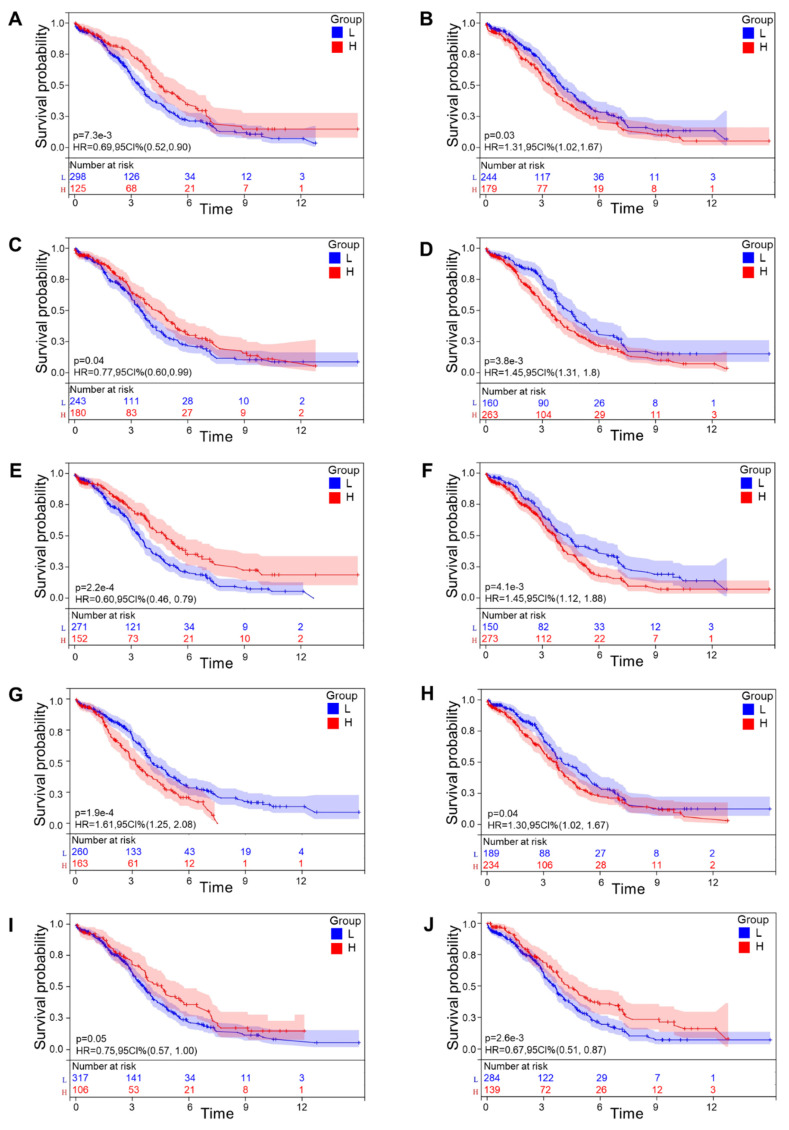
** Individual prognostic significance of genes**. Ten genes had relatively significant clinical prognostic ability (P<0.05). **(A)** PUM3,** (B)** KYAT1, **(C)** AMT, **(D)** REPS1, **(E)** CD40LG, **(F)** CXCR2, **(G)** AGFG1, **(H)** FGF23, **(I)** AHSG, **(J)** HAAO. Survival curves were generated using the Kaplan-Meier method. p value was calculated based on log-rank test.

Then a multivariate Cox regression analysis was carried out. Setting p < 0.05 as our screening criteria, 11 CRGs were ultimately obtained, including Pumilio RNA Binding Family Member 3 (PUM3), Kynurenine Aminotransferase 1 (KYAT1), Myosin Light Chain 2 (MYL2), Aminomethyltransferase (AMT), RALBP1 Associated Eps Domain Containing 1 (REPS1), CD40 Ligand (CD40LG), C-X-C Motif Chemokine Receptor 2 (CXCR2), ArfGAP with FG Repeats 1 (AGFG1), Fibroblast Growth Factor 23 (FGF23), Alpha 2-HS Glycoprotein (AHSG), and 3-Hydroxyanthranilate 3,4-Dioxygenase (HAAO). In an effort to make our results more legible, a forest map of these 11 CRGs was plotted (**Fig. 2A, B**). As all OV samples were separated into low- and high-risk categories, we displayed the Kaplan-Meier curve (**Fig. 2C**) and ROC curves at years 1, 3, and 5 (**Fig. 2D**). The AUC at 1, 3, and 5 years showed values of 0.75, 0.83, and 0.87, which were all greater than 0.75 and illustrated the exceptional accuracy and usefulness of the analytical procedure. The relationship among patients' risk scores, distribution of survival time, and gene expression was analyzed to further verify the prognostic model. As expected, we could recognize that the patient's survival rate declined dramatically as the risk score increased (**Fig. 2E**). On the basis of the subgroups of risk scores, we drew the Kaplan-Meier curves for these 11 CRGs that were eventually screened out, among which 10 genes showed relatively significant relationship with survival rate, including PUM3, KYAT1, AMT, REPS1, CD40LG, CXCR2, AGFG1, FGF23, AHSG, and HAAO (*p*<0.05) (**Fig. 3A-J**). These results showed that the model demonstrated excellent predictive accuracy and reliability.

### Construction and verification of nomogram of prediction model

**Figure 4:**
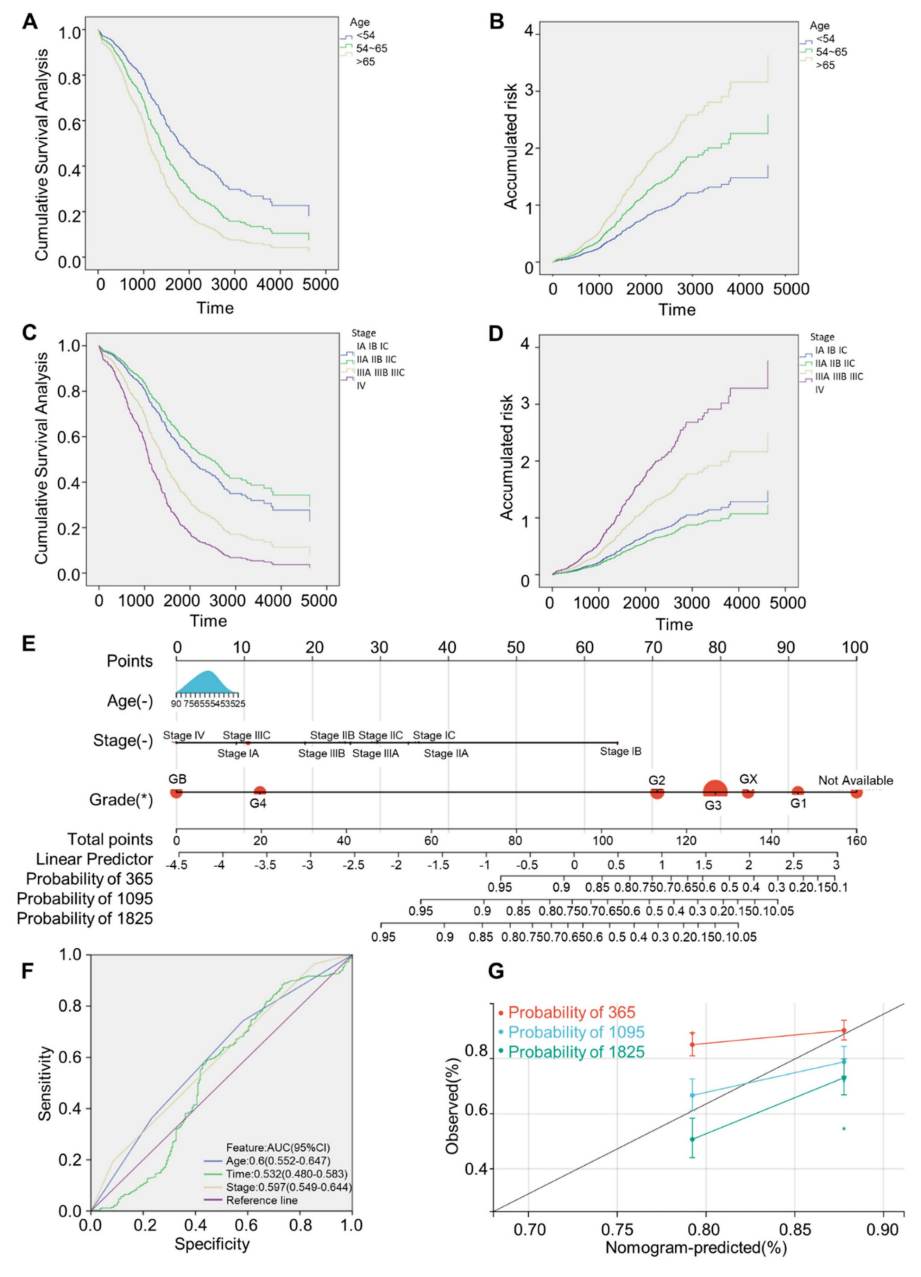
** Construction and verification of predictive nomogram model. (A)** Cumulative survival analysis curves grouped by age. **(B)** Cumulative risk profile by age. **(C)** Cumulative survival analysis curves grouped by FIGO stage. **(D)** Cumulative risk profile by FIGO stage. **(E)** 1, 3, 5 years survival nomogram. **(F)** ROC analysis curve. **(G)** Calibration curves of nomograms predicting 1-,3-and 5-year survival rates. Survival curves were generated using the Kaplan-Meier method. p value was calculated based on log-rank test.

Univariate Cox regression analysis of OV patients indicated that age and FIGO stage were strongly relevant to survival (both P < 0.05), as shown in Table 1. Further inclusion of the above high-related variables, age and FIGO stage, in the multivariate Cox regression analysis demonstrated that patients with age of onset > 65 years and FIGO stage IIIA-IV had a greater risk (**Fig. 4A-D**). By integrating the independent prognostic factors from the multivariate Cox regression analysis, R programming language was used to draw a nomogram model that can forecast survival rates of OV patients. Each influencing factor was projected onto the scoring table to obtain the score for each item, and then the scores were added up to obtain a final score. Specific survival rates of patients can be obtained by referring to the total score scale (**Fig. 4E**), and a higher total score means a lower survival probability for patients. ROC curves clearly illustrated nomogram prediction model had good discrimination, and the AUC values at age, time and FIGO stage were 0.6, 0.532, 0.597, respectively (**Fig. 4F**). The calibration curve was close to the black dotted line in an ideal case (**Fig. 4G**), indicating that the accuracy of the prediction was reliable.

### Differential expression analysis and functional enrichment of CRGs

**Figure 5:**
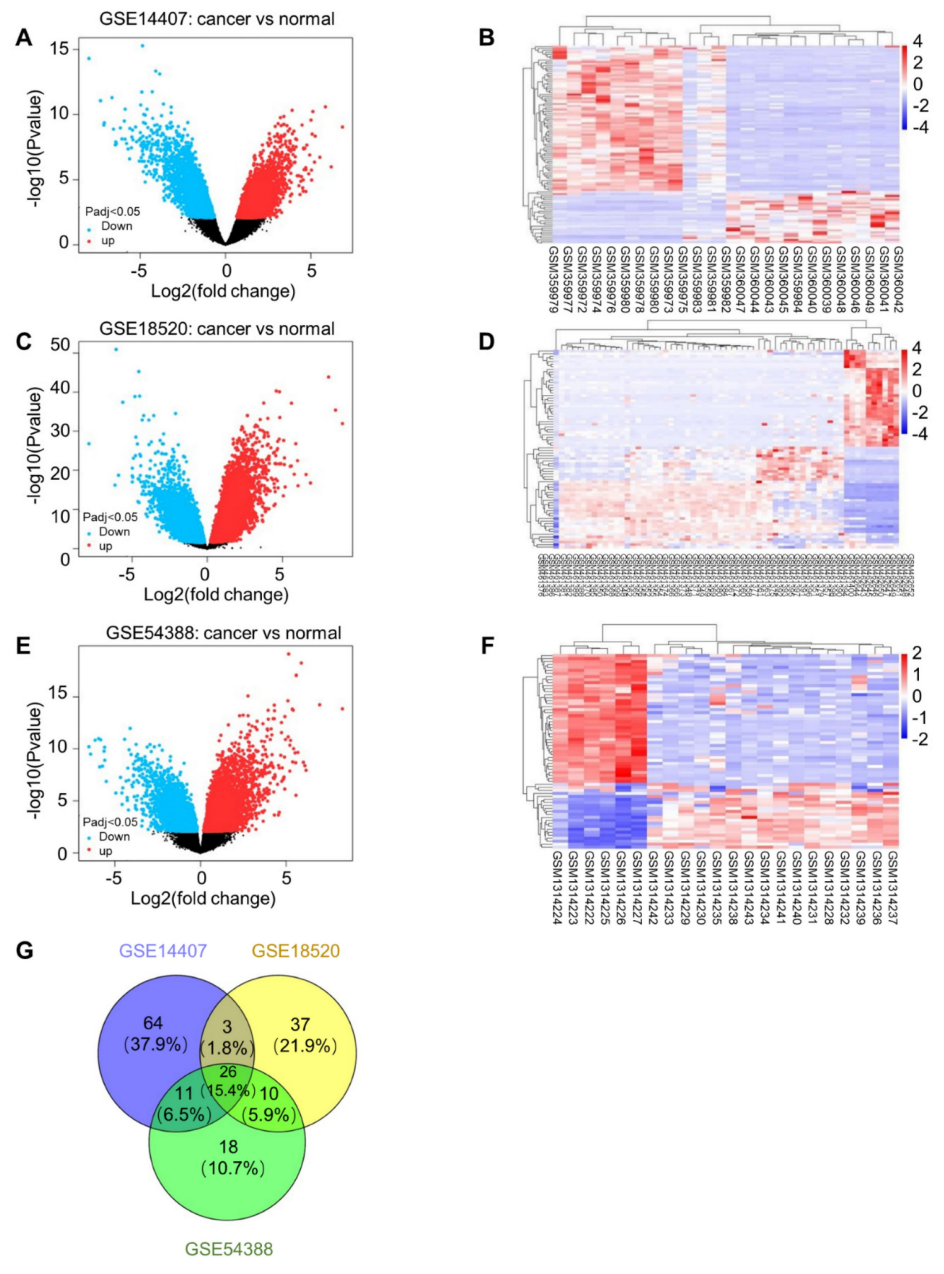
** Differential expression analysis. (A)** Differential gene volcanic map in dataset GSE14407. **(B)** Differential gene heat map in dataset GSE14407. **(C)** Differential gene volcanic map in dataset GSE18520. **(D)** Differential gene heat map in dataset GSE18520. **(E)** Differential gene volcanic map in dataset GSE54388. **(F)** Differential gene heat map in dataset GSE54388. **(G)** Venn diagram of differential gene in dataset GSE14407, GSE18520 and GSE54388.

A total of 167 common differentially expressed genes (P < 0.01 after correction, differential expression multiple |logFC| ≥ 4) were screened out from GSE14407, GSE18520 and GSE54388, including 65 up-regulated genes and 102 down-regulated genes (S3 Table). The differential gene volcano plot and heatmap of dataset GSE14407 (**Fig. 5A, B**), dataset GSE18520 (**Fig. 5C, D**) or dataset GSE54388 (**Fig. 5E, F**) are shown, respectively. Moreover, the Venn diagram of the three datasets is also shown (**Fig. 5G**).

**Figure 6:**
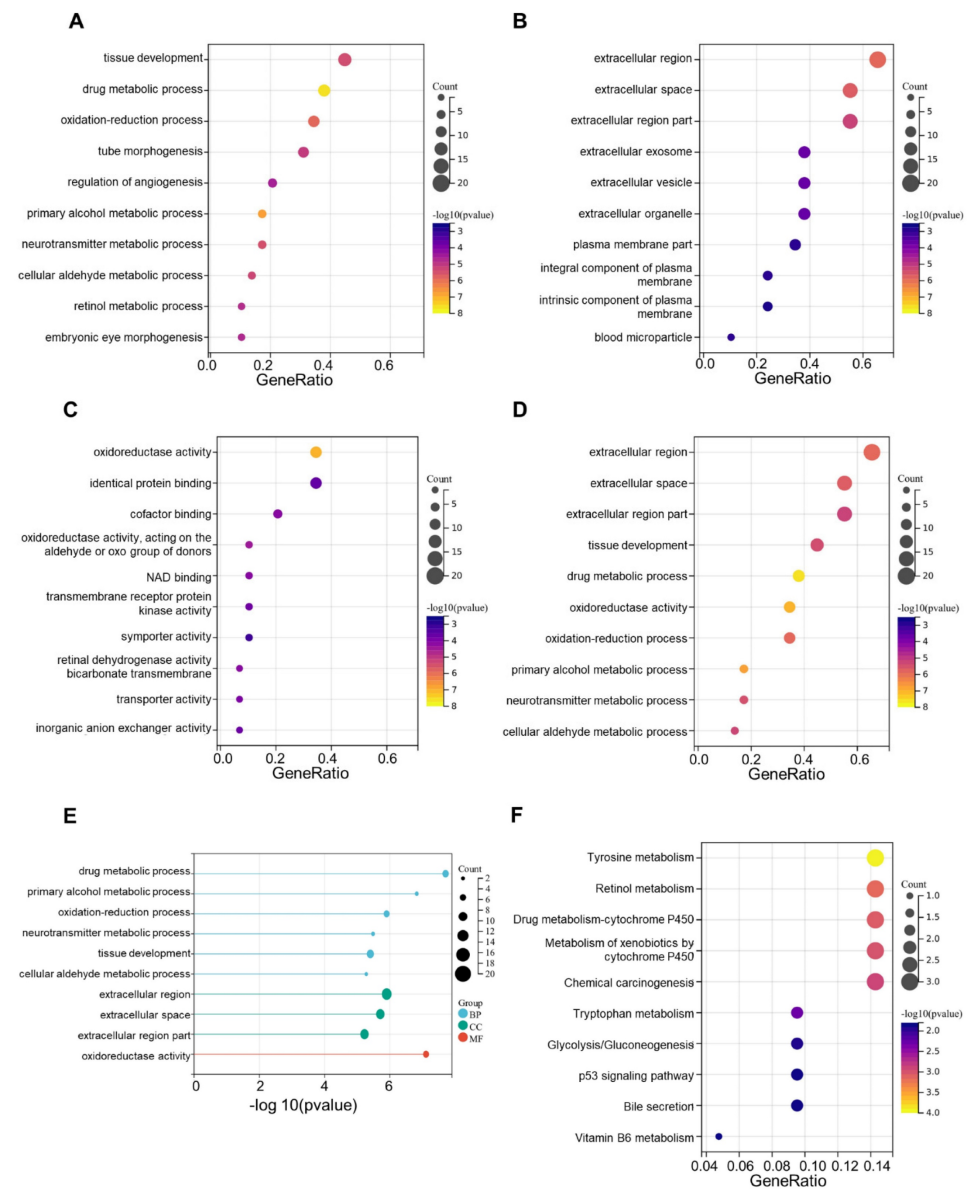
** GO enrichment and KEGG pathways analysis. (A)** The top 10 significant enriched terms in biological process (GO BP). **(B)** The top 10 significant enriched terms in cellular component (GO CC). **(C)** The top 10 significant enriched terms in molecular function (GO MF). **(D, E)** The top 10 significant enriched terms in three ontologies including BP, CC and MF. **(F)** The top 10 significant enriched pathways in KEGG analysis.

We then carried out an enrichment analysis using the Gene Ontology (GO) and Kyoto Encyclopedia of Genes and Genomes (KEGG) pathways. The result of gene ontology enrichment analysis showed that these CRGs were primarily concentrated in drug metabolism progress as well as tissue development (ontology: participating biological process, BP, **Fig. 6A**), extracellular region and extracellular space (ontology: cellular component, CC, **Fig. 6B**), and oxidoreductase activity (ontology: molecular function, MF, **Fig. 6C**). Meanwhile, in the KEGG analysis, CRGs that had differential expression in OV were primarily involved in tyrosine metabolism and retinol metabolism (**Fig. 6D-F**). The differential expression and enrichment analyses have identified key proteins and pathways involved in ovarian cancer development, but further in-depth analysis is required to elucidate the underlying mechanisms.

### Protein-protein interaction (PPI) networks analysis

**Figure 7:**
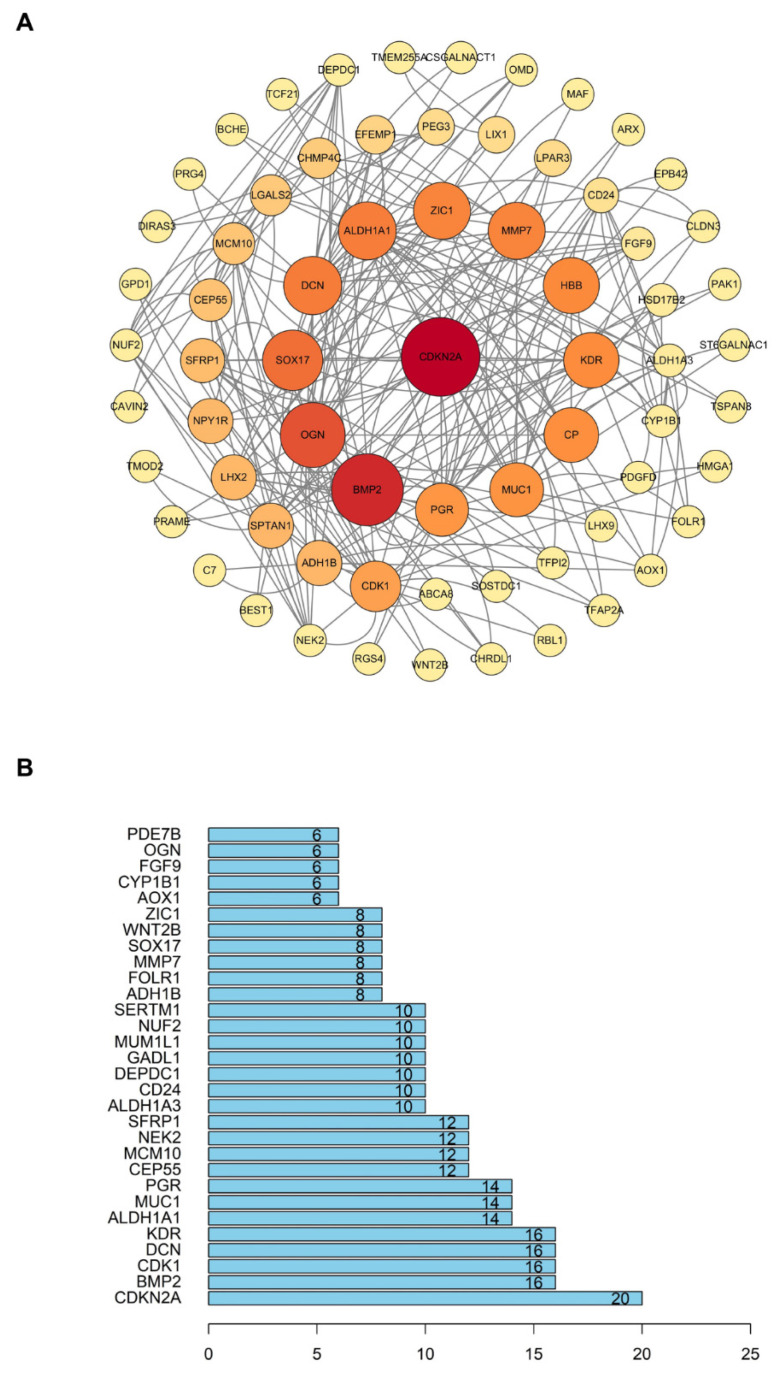
** PPI (Protein-protein interaction) network analysis. (A)** The PPI network constructed with 167 differentially expressed genes. **(B)** The top 30 genes with the most interactions.

In order to investigate the relationship between proteins encoded by 167 different expression genes, we imported these genes into the STRING database. After setting the interaction scores > 0.400, the protein-protein interaction networks were constructed, with an enrichment P value of 2.83e-14. The networks included 155 nodes and 126 interactions (Fig. 7A). After that, we calculated the number of interactions between each gene and visualized the top 30 genes with the most interactions using the R Programming Language. On this ground, the core gene of protein-protein interaction network was found to be CDKN2A (Fig. 7B). The results indicated that CDKN2A is likely a crucial factor in the development and progression of ovarian cancer.

### Expression and effect of CDKN2A in cuproptosis of OV

**Figure 8:**
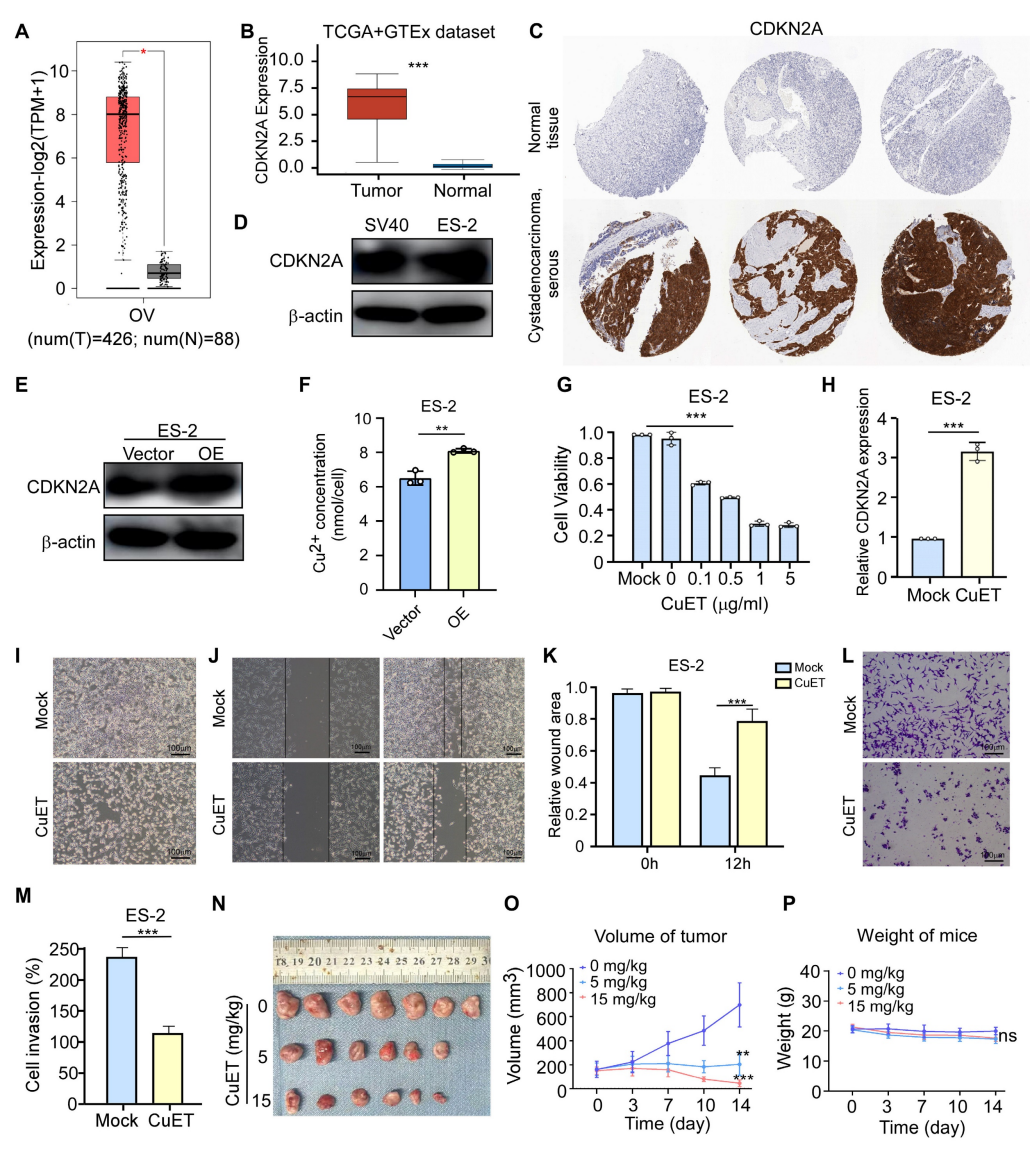
**Expression and influence of CDKN2A in cuproptosis of OV. (A)** Expression levels of group was imaged at 0 and 12 hours using a microscope. **(K)** The recover areas were calculated by ImageJ software in different groups at different time point. **(L)** Cell migration assay results of ES-2 cells in the control group and CuET treated group (0.5 μg/mL). **(M)** Calculate the degree of cell migration in the control group and CuET treated group (0.5 μg/mL) using ImageJ software. **(N, O)** Tumor size **(N)** and volume **(O)** measurements in the CDX model following CuET treatment (5mg/kg and 15mg/kg). **(P)** Body weights of mice across different treatment groups. n = 3, independent experiments **(D, E, F, G, H, K, L and M)**. Data presented as mean ± S.D. Statistical significance was assessed using Student's unpaired t-test. *p < 0.05, **p < 0.01, **p < 0.001.

CDKN2A (cyclin dependent kinase inhibitor 2A) located within the frequently deleted chromosomal region 9 of p21, is a tumor suppressor gene that plays an important role in cell cycle regulation 47, 48. To investigate the role of CDKN2A in OV, we analyzed the data sourced from the GEPIA website, which revealed significantly elevated CDKN2A expression in OV tissues compared to normal tissues (**Fig. 8A**). Complementary analysis of the TCGA and GTEx datasets confirmed these findings, demonstrating high CDKN2A expression in OV specimens (**Fig. 8B**). We furthermore analyzed the expression of CDKN2A using immunohistochemical staining from the Human Protein Atlas database. The results showed that CDKN2A is highly expressed in OV (**Fig. 8C**). To verify the expression level of CDKN2A in OV, we performed the Western blot experiment, which showed high CDKN2A expression in OV cell line (**Fig. 8D**).

We then explored whether CDKN2A can influence the progression of OV by regulating cuproptosis. Firstly, transient transfection of ES-2 cells with a CDKN2A-encoding plasmid resulted in successful CDKN2A protein overexpression (**Fig. 8E**), which in turn markedly increased the concentration of Cu^2+^ in OV cell line (**Fig. 8F**). To further investigate the function of CDKN2A in cuproptosis of OV, we first constructed a cuproptosis cellular model using ES-2 cells. Copper (II) Diethyldithiocarbamate (CuET) is a potent anticancer agent that has been proven to induce cuproptosis in tumor cells 49, 50. Therefore, the different concentrations of CuET were added to ES-2 cells, and then the MTT assay showed that the increasing concentration of CuET resulted in a continuous decrease in cell viability (**Fig. 8G**). Furthermore, we measured the mRNA expression levels of CDKN2A through qRT-PCR and found that it showed a significant increase after CuET treatment (**Fig. 8H**). Moreover, the bright images (**Fig. 8I**), wound healing assay (**Fig. 8J, K**) and transwell experiment (**Fig. 8L, M**) showed that cuproptosis induced by CuET could obviously suppress the cell viability, cell migration and invasion ability. To further analyzed the effects of CuET on OV, we utilized a cell-derived xenograft (CDX) mouse model to assess its anti-tumor efficacy *in vivo*. Compared to the control group, CuET treatment resulted in a significant reduction in tumor volume (**Fig. 8N-O**), with no substantial change in the body weights of mice across the different treatment groups (**Fig. 8P**). These results suggested that CDKN2A is highly expressed in OV, and cuproptosis could inhibit OV progression *in vivo* and *vitro*.

## Discussion

OV is the third highest annual incidence among female reproductive system cancers in China. The differential expression of CRGs and the prognostic model of CRGs in OV are of certain significance for the study, clinical assessment, and therapeutic method of OV, which may decrease the mortality possibility of OV patients, improve the survival rate, and achieve "early prevention, early detection, and early treatment" in as far as is possible 51.

In this study, there were 62 CRGs sequentially covered employing univariate Cox regression analysis as well as LASSO regression analysis stepwise. What's more, we investigated the differential expressions of CRGs in OV thoroughly. After the risk model was constructed, we curved the Kaplan-Meier curve and ROC curves of 1, 3, and 5 years, with the intention of analysis. The analysis results indicated that CRGs had potential value and effect in OV. It is of certain authenticity and feasibility that these CRGs can be used in the construction of prognosis pattern. Eleven CRGs (PUM3, KYAT1, MYL2, AMT, REPS1, CD40LG, CXCR2, AGFG1, FGF23, AHSG, and HAAO) were recognized as multivariate Cox regression analysis and a prognostic pattern for CRGs in OV was constructed. Given that CD40LG is a prognostic marker related to immunity and stroma in the breast cancer tumor microenvironment in the study of breast cancer 52, this study enables us to draw lessons from and apply the research's ideas and methods, thus we can be much more adequately prepared for the subsequent investigation of the cuproptosis-related gene CD40LG in OV. The precise connection between the AHSG gene and OV can be further investigated. Previous research has shown that ovarian endometriosis cysts in women of childbearing age are associated with ovarian AHSG gene polymorphism, and OV is also associated with ovarian endometriosis cysts.

Differentially expressed CRGs in OV were functionally enriched utilizing GO and KEGG, which determined that these CRGs were predominantly abundant in drug metabolism and primary ethanol metabolism, oxidoreductase activity, tyrosine metabolism and retinol metabolism. The aforementioned metabolic processes and enzyme activity have a certain correlation to the appearance and progression of OV. Many studies have recognized the importance of drug metabolism-related genes in the treatment and prognosis of OV. Patients with high expression of genes related to drug metabolism have good prognosis 53. In addition, immune-infiltration analysis also indicated that abnormally expressed genes related to drug metabolism may play an important role in the regulation of infiltration of macrophages and neutrophils in OV tissue. What's more, the accumulation of acetaldehyde, a derivative of ethanol metabolism, is closely associated with the production of free radicals, the decrease in glutathione (GSH) levels, and the formation of protein and DNA adduct compounds, which may adversely affect key biological functions, thereby promoting pathological changes in cells and tissues, and leading to the development of cancer 54. In addition, nicotinamide adenine dinucleotide phosphate (NADPH) homeostasis is regulated by multiple signaling pathways and several metabolic enzymes that undergo adaptive alterations in cancer cells. The metabolic reprogramming of NADPH makes cancer cells highly dependent on this metabolic network for antioxidant capacity and more susceptible to oxidative stress 55. In our study, several CRGs identified in ovarian cancer, including FGF23 56 and AGFG1 57, are involved in drug metabolism. Given their roles, therapy status could influence the expression of these genes and the observed differences. Our study has limitations, particularly the lack of a comprehensive analysis of therapy status and its potential effects on gene expression. Future research can address these limitations through more in-depth analyses.

Moreover, our study identified CDKN2A to be the core gene through the building of protein-protein interaction (PPI) networks. It is crucial for life activities such as cell cycle regulation, gene expression control, biological signal transmission, and energy and material metabolism, which are all closely related to the development of tumors 58. In some cancers, the high expression of CDKN2A is associated with poor prognosis. For example, in colon adenocarcinoma (COAD), bladder urothelial carcinoma (BLCA), and liver hepatocellular carcinoma (LIHC), the high expression of CDKN2A is correlated with poor prognosis in patients. However, in other cancers, the loss or low expression of CDKN2A may indicate a worse prognosis. Although, previous studies have indeed explored aspects of cuproptosis mechanisms in ovarian cancer 59, they did not specifically highlight the importance of the cuproptosis-related gene CDKN2A in ovarian cancer. In contrast, we demonstrated that the expression level of CDKN2A increases in cuproptosis of OV by the *in vitro* functional assays which may inhibit viability as well as migration and invasion ability of OV cells. However, the CDKN2A gene is not included in the CRGs prognostic model for OV, which may be attributed to that the current high-throughput data and clinical sample information are not comprehensive enough, or our prognostic model needs further optimization. Moreover, the main limitation of our research is that our results are mainly based on big data analysis of biological information, so more experiments are needed to verify the function of CDKN2A gene in the occurrence and development of OV, and to further reveal its potential mechanism in cuproptosis in OV.

In conclusion, we have developed a unique prognostic model for genes associated with cuproptosis in OV. Future research is expected to concentrate on the mechanisms underlying the genes that trigger cuproptosis in OV in order to provide an alternative clinical approach for curing the sickness.

## Supplementary Material

Supplementary figure and tables.

## Figures and Tables

**Table 1:** One-way cox regression analysis.

Characteristics	RC	Wald	Degrees of freedom	P-value^a^	Exp (RC) 95%CI
Age	0.351	25.573	1	0	1.420 (1.240-1.627)
FIGO Stage	0.361	11.151	1	0.001	1.435 (1.161-1.773)
Grade	0.210	2.018	1	0.155	1.234 (0.923-1.649)

RC, Regression coefficient; CI, confidence interval; FIGO, Federation International of Gynecology and Obstetrics. ^a^P-values were calculated using the Cox-proportional hazard model.

## Abbreviations

TCA: tricarboxylic acid
TCGA: The Cancer Genome Atlas
KEGG: Kyoto Encyclopedia of Genes and Genomes
GO: Gene Ontology
PPI: protein-protein interaction
ROS: reactive oxygen species
CRG: cell death-related gene
OV: ovarian cancer
