# Supplementary material for: Implication of CDKN2A in cuproptosis through defining cuproptosis-related gene signature in ovarian cancer
Source: J Cancer. 2025 Jul 24;16(11):3355–71. doi: 10.7150/jca.115374 (PMC12374941; doi:10.7150/jca.115374)
Supplement: Supplementary file 1 — Supplementary figure and tables. [file jcav16p3355s1.pdf]

Supporting information

Supplementary Table S1. 200 cuproptosis-related genes and related data

Gene	HR	HR.95L	HR.95H	pvalue
NPY	1.11976870526294	1.03166418507081	1.21539738553604	0.00681954152407706
OTC	0.646166414149701	0.439156757610253	0.950756256256081	0.0266751726827593
AHSG	0.382325680360714	0.151680274587241	0.963691068341315	0.0415127177377878
ACE2	0.0791879896566677	0.00774329699232333	0.80982786945681	0.0325345867041235
SLC22A2	9.70078468344863	2.13066069285468	44.167156126839	0.00330268496712338
FGF23	12.5228221437618	3.19607972131305	49.0666967405504	0.000286099602484026
ARG1	1.75982703968154	1.01728127963248	3.0443804202444	0.0432554787537694
GAD1	0.695013079001275	0.490448498076486	0.984900926146789	0.0408054224407593
MGAM	3.37766444657966	1.3122602421073	8.69386783780609	0.0116250250613567
VIP	2.37205883628025	1.00767658545208	5.58379861555572	0.0479883592235405
SLC38A4	1.40419147207762	1.05655236118549	1.86621483486454	0.0193349231622828
MYL2	2.7962842855789	1.58263270589583	4.94063200933887	0.000398956517832606
KYAT1	8.39329533511061	1.45330635188466	48.4738861087566	0.0174162317635705
HPR	2.06907517707373	1.0869967227404	3.93844065839476	0.0268310512706729
PON3	0.826184842593815	0.697688817665875	0.978346473167435	0.0268439374405578
BHMT2	1.20824942536286	1.00219503404054	1.45666923533231	0.0473732840851792
AMT	0.0829640931199976	0.00836559130486941	0.822779944224288	0.0334528437892755
HPGD	0.914289257245474	0.837768827374843	0.997798937606521	0.0444973818806958
GAP43	1.29934990159365	1.0254855636433	1.64635195913755	0.0301319491216501

CXCR2	1.90086003505675	1.35757256689442	2.66156591624538	0.000184057697030572
REN	0.854914507996732	0.750785145741884	0.97348598347811	0.0180072210828748
CACNA1C	2.04666678899952	1.45198019759783	2.88491878341293	4.32902443717489e-05
LEPR	1.26606571179019	1.01091630149551	1.58561335315249	0.0399257677408219
ARSH	0.564669399908401	0.371350468036873	0.858626980810089	0.00752244656886092
MAPT	1.66535571551736	1.05977272686662	2.61698531099809	0.0269867940267938
MEOX2	1.23139466760201	1.00910118695301	1.50265686633195	0.0404467110767418
PYY	0.897031566521411	0.80608878135943	0.99823449965254	0.0463312312249476
HGF	1.48658981555107	1.09548985677792	2.01731605822452	0.0109123814239993
MADCAM1	0.469875589007301	0.236254101755766	0.934515284620109	0.0313164824219374
LYVE1	1.26638015738035	1.10389437584702	1.45278274633488	0.000749549723496998
SYNGAP1-AS1	0.495885154262302	0.280614968183722	0.876297112051242	0.0157554264684958
ITGBL1	1.29613305405798	1.01455421552733	1.65586113399418	0.0379321972947897
IFNG	0.574097250600147	0.358566300153984	0.919181900265331	0.0208397637358684
SLC7A11	0.79270089293611	0.670009396957747	0.937859541246602	0.00677431250082483
SCN4B	1.30505674516109	1.00382372625998	1.69668544739034	0.0467600546244711
PTGER3	1.16454621727743	1.00140680571417	1.35426270765954	0.047903856603669
MS4A1	0.601080121804873	0.392033774088055	0.921597415093655	0.0195750047683569
CETP	0.477518505723661	0.280901171909453	0.811758533289637	0.00632713566684319
SLC6A12	1.14201882590728	1.00334833594192	1.29985464868718	0.0443705189543664
CD40LG	0.41781562130912	0.258882917132358	0.674319863757846	0.000352288916458301
PAOX	0.482020094740	0.2629486223897	0.88360748811	0.0182669839978

	094	09	5678	662
FLNC	1.11897060626499	1.0055387869609 3	1.24519832941431	0.0392793319688067
NGFR	1.14173686826031	1.0162231721454 4	1.28275275753927	0.025693964573537
LOXL4	1.14344473891419	1.0232863767201 5	1.27771257459837	0.0179654379416086
APC	1.53264909836585	1.0862185425395 4	2.16256044868257	0.0150670738188632
IGLL1	0.639732535070354	0.431018577431826	0.949512939479445	0.0266170579842385
CMC2	0.611765754754862	0.388172885429006	0.964151162380027	0.0342379679231138
PTGER2	1.18570786659111	1.0583675389285 4	1.3283694871437	0.00329706706923022
NEFL	1.52277352830002	1.1530645559994 6	2.01102289236648	0.0030398135605498
PLA2G4A	0.870200130727732	0.767313789261887	0.986882131034021	0.0303381008993
VPS33B	0.519980432428806	0.339375030451134	0.796698713365653	0.00266489940608113
CTLA4	0.692771821055916	0.508329032798782	0.944138078060767	0.0201308511521211
HAAO	0.744847778729429	0.584222901854811	0.949634483202853	0.0174559690991748
JAK2	0.677701360845064	0.4963749755434 5	0.925266496338609	0.0143299958059948
ACSM3	0.77020059729566	0.651932156552216	0.909924374971492	0.00214257605770186
CD274	0.694378491797608	0.516943723028344	0.932715629172431	0.0154091617797908
RYR1	1.19715480965001	1.0247282052255 7	1.39859489663667	0.0233405814408213
FNTB	0.652064958419658	0.449017107875808	0.946932093546048	0.024676983854198
PGM3	0.71770896443809	0.5604614010123 9	0.919075170394131	0.0085696391300501
ABCC3	1.19175784931124	1.0247574378489 2	1.38597361574296	0.0227590528053139
COG3	1.49368665641554	1.1408796754166 9	1.95559608574756	0.00351522540032151
CFI	0.815720313772708	0.705243509720033	0.943503373133622	0.00608445210513006
PPARA	1.367926541826	1.051104363604	1.78024474888	0.0197661472786

	99		369	854
SLC35A1	0.740662677552561	0.594935401465029	0.922085323160208	0.00723937420325936
ALPL	0.904825887370149	0.842004038774714	0.972334868662347	0.00644733522616211
ALOX12	1.48834110441589	1.15368942076364	1.92006549009324	0.00221249340164654
SLAIN1	0.863438629101938	0.748418721083736	0.996135245181849	0.0441091728648245
COL8A2	1.11937112826745	1.00226414473501	1.25016117695204	0.0454920289022673
SLC33A1	0.696200920058263	0.505270531315117	0.959279615671247	0.0268178036733941
SLC16A1	0.868074462856797	0.754290851992457	0.999022155808477	0.0484259282042127
CDC7	0.787397281408187	0.658059104689947	0.942156220239703	0.00903336198404115
REPS1	1.43418372168728	1.10801028527379	1.85637531969708	0.00616185538444921
PRSS1	0.875553756494338	0.802670759514675	0.955054574275084	0.00272633683646723
RXRA	1.24624407115356	1.01637395389677	1.52810319364318	0.0343373783203344
SNX14	0.764272009224524	0.594674381580753	0.982237880386598	0.0357324876269968
TRPM2	1.25823140230775	1.02392772018499	1.54615040744019	0.0288980964238435
BTRC	1.37250509300323	1.03688170940548	1.81676483752415	0.0268904207325276
IGF2R	1.3397898418917	1.10232698723394	1.62840685316111	0.00329562875377947
UFD1	0.687964370542759	0.511630135357021	0.925072513185769	0.0133075085334419
TIMM8A	0.693127181021383	0.512514444392269	0.93738877865253	0.0173245776755062
PTRH2	0.734364883907057	0.578279791936043	0.932579333111938	0.0113250878098757
GGCX	1.39518878541189	1.03434558336498	1.88191623597068	0.0291739041239842
NPC1	1.29329162870802	1.03740790547459	1.61229081449987	0.022227627705972
PTPRU	1.20653944086594	1.06212290293201	1.37059225288006	0.00389500732970857
MVK	1.407987551776	1.0904943802935	1.81791761771	0.0086791374190

	31	4	704	7542
BACE1	0.822653649234064	0.678204530926721	0.997868630681924	0.0475215642706914
AP1S2	0.734748729918648	0.622106756378103	0.867786261091422	0.000283289176441682
SLC4A11	1.12770830367941	1.0237187120325	1.24226118292064	0.0148971653932335
SELL	0.821886515677261	0.715272696948584	0.944391485280845	0.00565622705899917
GARS1	0.760456710977074	0.595225807910346	0.971554663095476	0.0284632202175311
AGFG1	1.4651325245132	1.05960598560906	2.02585993618425	0.0208800208020345
PPP3CA	1.33652457966629	1.0821608296298	1.65067696329688	0.00708071666958585
PNP	0.857550562207826	0.738795002542317	0.995395155912464	0.0433157756352683
RIN2	1.38199702787532	1.10965733756407	1.72117618691991	0.00386281685782643
CDC45	0.824957681895149	0.690757311957439	0.985230507353284	0.0336512908593138
CD38	0.682840575264824	0.55194981238094	0.844771101953356	0.000442038274401555
GRIN2D	1.17446531379588	1.03432367045924	1.33359490138829	0.0131191454071061
PDP1	1.39722100629501	1.1470893271858	1.70189582813181	0.000889057678792234
ANXA4	1.21296854700751	1.02449674191457	1.43611261591713	0.0250353743681789
HIF1AN	1.44826865418346	1.10552324791427	1.89727542921199	0.00718694664918385
RPS6KA1	1.22373424660395	1.01029839963176	1.48226059435229	0.0389477271121984
PSMD6	0.716810251239268	0.517585427223255	0.992719093808786	0.0450757251668758
GCH1	0.758307041955455	0.621517623008019	0.925202357249672	0.00641167040648986
AUH	1.32256980844965	1.0511113977452	1.66413465021389	0.0170675019142477
XDH	1.20496859429774	1.02526565889012	1.41616887355384	0.023649547365998
FOXO1	1.19297508343589	1.00217080149803	1.42010677977397	0.0472168086708541
DCAF8	0.757340433266	0.5748419540969	0.99777778530	0.0481798838723

	605	78	7031	307
PER1	1.21003817831117	1.03221442027264	1.41849635522807	0.0187261926360312
CYBRD1	1.18730516800462	1.04929767240942	1.34346391785422	0.0064643100526284
PXN	1.29304701451406	1.04236685292046	1.60401357454842	0.0194216866976987
GJB1	0.881161587804294	0.796771569640707	0.974489770226004	0.0137757815687253
BLOC1S1	0.762737123031586	0.651460951296063	0.893020399293451	0.000762043699945739
CASP8	0.747379161081661	0.596605811297974	0.936255731743365	0.0113116712894034
STAB1	1.15983035616982	1.02529580454071	1.31201790657439	0.0184189911172438
PPP1R15A	1.21118864646094	1.0230822309183	1.43388077026726	0.0260841378051008
GAS1	1.12204563581005	1.01685510216803	1.2381178067122	0.0218618152597547
UQCC2	0.79937054724512	0.676458083898635	0.944616210542193	0.00856760544493223
CASP3	0.78086601347242	0.622675472739908	0.979244819638181	0.0322295700447598
TXNL1	0.734044139042645	0.552565371258407	0.975125887523049	0.0328591653264657
HSPA14	0.746295551013838	0.591178568524601	0.942113058754889	0.0138335968000052
SLC1A4	0.792431681164042	0.662588972098995	0.947718715153398	0.0108315856743075
ELOC	0.79898393141569	0.647507203581036	0.985896865903484	0.0364032131742781
LRP1	1.16170693210624	1.01752832003241	1.32631492365804	0.0266249477672665
DDIT3	0.825610974147039	0.684595006471903	0.995674046973393	0.0449276543841792
ELAVL1	0.618842458714552	0.437056876816682	0.876238331947131	0.00684045286238236
NME1	0.840138670016116	0.721347602323981	0.978492175731161	0.0251232331302039
LTA4H	1.27627698450578	1.03672527330119	1.57118089346119	0.0214473520914038
TDP2	0.663458019016797	0.511594217948648	0.860401715958948	0.00197688031167535
COA6	0.772796902683	0.6341820221122	0.94170921277	0.0106054999677

	517	52	1611	167
GMNN	0.778882407117079	0.663677104262391	0.914085780902045	0.00221389110969623
COMMD4	0.726019564404651	0.57861345155736	0.910978489144348	0.0056890164526533
IDO1	0.898877052154686	0.825920909842831	0.978277635620162	0.0135689155308505
ECE1	1.174637043948	1.00014535996733	1.37957165052485	0.0497932898445681
PYM1	0.749928632590249	0.562768910069542	0.99933195298437	0.0494691281322418
ERBB2	1.2172657891984	1.05390597575218	1.40594705376376	0.00749384184492692
CRYAB	1.08752755191254	1.00654165442503	1.17502953898563	0.0335778820705145
MBD2	0.758942922413043	0.603100480024281	0.955055382243536	0.0186674979907553
ADI1	0.740498330345587	0.587956199256651	0.932616711819455	0.0106888814933814
TGFBI	1.13180186826929	1.01738180510012	1.25909020841177	0.0227941790870418
H6PD	1.22593335255128	1.04580300805157	1.43708956020093	0.011994452659779
APEX2	0.711986952708617	0.552427534067142	0.917632430619742	0.00869192812027321
PUM3	0.768543177836229	0.625754598186833	0.943914144474661	0.0120605316477975
KCTD1	0.830597440956547	0.716853607151178	0.962389115492129	0.0135053646427027
DCTN1	1.29198052275705	1.03332144660649	1.61538665113882	0.0246054183682035
DAXX	0.748306961496497	0.579232419199517	0.966733369996752	0.0264949585207416
CDKN1B	1.20529318939413	1.00368247674446	1.44740164948574	0.0455735884015321
RANBP1	0.77830937310192	0.631411489059278	0.959383050126024	0.0188489629327475
HPS1	1.34320316982109	1.07138686699042	1.68398065255877	0.0105370697613381
GLUD1	1.25159116670257	1.00323653361225	1.56142683812325	0.046745667578476
NAPRT	0.870070772542715	0.76832128260676	0.985295040461003	0.0282765109606951
UBE2L3	0.727011469220	0.5650470797143	0.93540112913	0.0131646189540

	465	33	3012	5
CIZ1	1.33194666410226	1.03828229302557	1.70867010631901	0.0240966599202849
FURIN	0.829902435941894	0.70154624437899	0.981742912460407	0.0296375755656131
CD79A	0.886056489703804	0.810514532381399	0.968639144124303	0.00779601907492652
RELB	1.19902304782689	1.00152681525895	1.43546457999566	0.0480893470687794
BIRC5	0.869205747332303	0.763200524212283	0.989934633463854	0.0346508194935292
FLOT2	1.22828308824035	1.00651710398172	1.49891078739647	0.0429800183894874
HNRNPA2B1	0.762276836249748	0.590784713234297	0.983549442066356	0.0368404593653766
GSTK1	0.805170246592548	0.665470339833124	0.974196875792353	0.0258244869658635
VCP	0.805526559061773	0.655047609456104	0.990573857513453	0.0403908015249926
FUS	0.743111440410071	0.563718268067101	0.979593254555659	0.0351849507740164
QPRT	0.859851931924443	0.753973838627491	0.980598141415725	0.0243095732778698
COX7A2	0.84828057544176	0.724293517949383	0.993492164211376	0.0412559068279048
COA4	0.758212084493557	0.622128411137579	0.924062548471096	0.00609823313274956
CITED2	1.31200511809911	1.13439950723424	1.51741729341461	0.000253010783241838
GLO1	0.782376877235659	0.641564711031752	0.954094836432996	0.0153450723264698
CALM1	0.728862012273275	0.585818366528784	0.906833693321811	0.00454959498903392
HSPD1	0.810988940183129	0.666662048188332	0.986561426267891	0.0361462616184046
CCL5	0.917747625701407	0.845803856120483	0.995810906258851	0.0393268303930542
STAT1	0.809971018812758	0.707729248955982	0.926983097398284	0.0022041341972013
TIMM23	0.709031573664128	0.559879101421152	0.897918445565393	0.00432367622868968
B4GALT5	1.24148901416351	1.02809911341089	1.49916963470105	0.0245795280463265
SSR4	0.844999819200	0.7262993977853	0.98309966471	0.0292088240952

	584	4	9882	279
CXCR4	0.853541985154689	0.758522602729355	0.960464352413968	0.00854215813916945
DPM3	0.805780754965564	0.701123474826631	0.926060313746341	0.00234926242829305
MORF4L2	0.702831933379093	0.524188597769747	0.942356870559734	0.0184353947009525
SUMO2	0.790517336625553	0.635533125189993	0.983296754702971	0.0347507854548949
CXCL9	0.885619096846026	0.824720972018957	0.951013992985183	0.000832470835837791
APEX1	0.793242083281077	0.632929911620365	0.994159054795186	0.0443445162383289
FLOT1	0.788121467918192	0.644093910054622	0.964355412304472	0.0207520178236296
CUTA	0.802529393269413	0.667339387085727	0.965106270549915	0.0194221597347851
CFL1	0.749037624145561	0.583776949105097	0.961081733778118	0.0230814005809312
IDH2	0.863462665026334	0.746189089372354	0.999167348482008	0.0487068730387143
XBP1	0.820519360035112	0.710628556572067	0.947403554171908	0.0070083592410475
IGFBP7	0.848908083750641	0.734188774764497	0.981552646168344	0.02701333054561
GNAS	0.715902377882403	0.573005110986599	0.894435677502475	0.00326059469964145
PRDX6	0.719725582592045	0.545398863968444	0.949772631479901	0.0201185745337149
ILF2	0.764935833117452	0.596681015013393	0.980635907736996	0.0344942232254913
CXCL10	0.918452442992486	0.863553282641361	0.976841738657601	0.00682935676053675
UCP2	0.85545480744382	0.750246994348292	0.975416007117002	0.0197153979940276
RPL21	1.18250913561101	1.00502908667885	1.39133073294855	0.0433394308841627
GSTP1	0.819659557046502	0.680336211519986	0.987514376100399	0.0364256222189967
HLA-A	0.880328985521096	0.781766875027402	0.991317421477391	0.0353864241416974
PRDX5	0.804293122251144	0.662094456502641	0.977031932750994	0.0282322300944595
TUBB	0.793075153813	0.6390852502372	0.98416948186	0.0353053977496

	178	2	8548	021
HSP90AA1	0.80509979300912	0.65915622750368	0.98335667578853	0.0336339166758037
TMSB4X	0.855471050322217	0.743714519605092	0.984021016999903	0.0288536547658368
HSP90AB1	0.768905876058768	0.613028584915693	0.964418724975131	0.0230026662645202
IGHG1	0.954184093463042	0.919985826919096	0.989653598541745	0.0117869079892719

Supplementary Table 2. 62 Cuproptosis-related Genes analyzed with LASSO Regression

Gene	Coef
NPY	0.0135732812109809
AHSG	-0.561271543765313
ACE2	-0.0385417588833998
SLC22A2	0.952557817484441
FGF23	1.1357462242778
GAD1	-0.0677094349641544
MYL2	0.62721981223141
KYAT1	1.97225786273736
HPR	0.120833790910917
BHMT2	0.0230058797303406
AMT	-2.00172577883352
CXCR2	0.345613453960716
REN	-0.0115013756468305
CACNA1C	0.476708349681969
ARSH	-0.273738610658473
MEOX2	0.0192229056037472
LYVE1	0.118630934682722
SLC7A11	-0.115291002992019
CETP	-0.189930537803133
CD40LG	-0.653447359825286
NEFL	0.098837615927497
PLA2G4A	-0.0552797434117821
HAAO	-0.180505456313912
ACSM3	-0.005689778044511
RYR1	0.045001986605304
FNTB	-0.065546147760714
PGM3	-0.0865849287445835
ALPL	-0.0385654390728529
ALOX12	0.0571395362743804
REPS1	0.238055996036934
SNX14	-0.0672583707368904
MVK	0.0616547722829491
BACE1	-0.0617772237838801
AP1S2	-0.00257309718246001
GARS1	-0.010684161404821
AGFG1	0.136617086217292
PPP3CA	0.0446553242487762
CD38	-0.0460042857158858
GRIN2D	0.0650548975820607
PDP1	0.143963833663349
AUH	0.0917213235704573

FOXO1	0.038846423205137
GAS1	0.037434134064215
TXNL1	-0.0418537375586716
ELAVL1	-0.0708971864853319
TDP2	-0.0129042576343187
ADI1	-0.0043067842799895
PUM3	-0.155356891091965
CDKN1B	0.119227370951847
NAPRT	-0.0213142378403041
FURIN	-0.0919208747332471
FLOT2	0.0302423678423873
GSTK1	-0.00948361046867879
GLO1	-0.102022014907769
CALM1	-0.162108793858512
DPM3	-0.0011548766200263
CXCL9	-0.0162106158515676
CUTA	-0.000416318814452632
IGFBP7	-0.0741769271520729
GNAS	-0.1553474206216
HSP90AB1	-0.0873477243586898

Supplementary Table S3. Co-differentially expressed Cuproptosis-related Genes

GSE14407	GSE54388	GSE18520
LRRN4	BNC1	BNC1
KLHL14	LINC01105	RTP3
SLC4A4	SLC4A4	LINC01105
LINC01105	PDE8B	SLC4A4
SLC26A7	KLHL14	SPIN3
BNC1	LOC100996760	CCDC60
MICALL2	CP	BEST1
LOC100996760	LHX9	KLHL14
CP	HBB	RBL1
ALDH1A3	ABCA8	HIF3A
NXPH2	MAF	NFIA-AS2
LHX9	EFEMP1	ZNF577
HBB	SFRP1	UBAC2-AS1
GADL1	SPOCK1	LINC00550
PCDH9	C7	CP
ABCA8	CDK1	MB21D1
CSGALNACT1	TSPAN8	LHX9
TXNIP	KDR	ANKH
EFEMP1	CLDN3	PRKCQ-AS1
DCN	MMP7	HBB
SFRP1	REEP1	GPD1
SPOCK1	FOLR1	C16orf45
NELL2	NEK2	PAK1
PRAME	MNDA	ABCA8
RGS4	AOX1	RNF187
REEP1	BCHE	EFEMP1
TFAP2A	NPY1R	SFRP1
HLF	SCGB2A1	SLC4A2
MAL	PRG4	SPOCK1
HSD17B2	LHX2	CYP1B1
GLDC	WNT2B	REEP1
AOX1	CLEC4M	TCF21
GFPT2	PTGIS	MNDA
RNASE4	MECOM	AOX1
BMP2	CD24	BCAP29
S100A1	PEG3	BCHE
SULT1C2	GPM6A	NPY1R
BCHE	ADH1B	SCGB2A1
AQP9	CHRDL1	HMGA1
OMD	TCEAL2	MEOX2
PPM1E	IGK	SLC5A1

PRG4	ALDH1A1	MECOM
DSC3	DIRAS3	SPTAN1
LHX2	Igk	CD24
MEOX2	C8orf4	GPM6A
ZIC1	CEP55	ADH1B
HSPB3	OGN	IGK
RARRES1	NDUFAF4	LOC647070
FGF9	PEX5L	DIRAS3
WNT2B	HAND2-AS1	Igk
PTH2R	EHF	MAGEF1
CDKN2A	SOX17	ZNF302
SGCG	LPAR3	C8orf4
MUC1	PSAT1	OGN
PGR	NUF2	NDUFAF4
TRPA1	ITLN1	RNF128
MECOM	MGARP	ELL3
LGALS2	CHMP4C	PEX5L
CD24	MUM1L1	HAND2-AS1
TFPI2	SHISA3	TMEM255A
SPON1	ARX	SOX17
GPM6A	C1orf168	LPAR3
ADH1B	Ndufaf4	ITLN1
SEL1L2	PRSS35	RTBDN
CHRDL1	SERTM1	MGARP
EPB42		PLEKHH2
TCEAL2		MIAT
IGK		TCEAL7
SOSTDC1		KIAA2026
Igk		DPP10
SDPR		SHISA3
OGN		DLX6-AS1
PDGFD		Ndufaf4
RAB38		PRSS35
PEX5L		CAPSL
TMOD2		LOC101060391
HAND2-AS1		
EHF		
TMEM255A		
MMP28		
SOX17		
DEPDC1		
PDE7B		
C21orf62		
MCM10		

ITLN1
MGARP
SLC4A11
STK31
ARHGAP18
TCEAL7
ST6GALNAC1
RERG
MUM1L1
ANKRD29
LIX1
ARX
C1orf168
KCNT2
UNC5A
CRNDE
LOC101928554
AADACL2
SERTM1

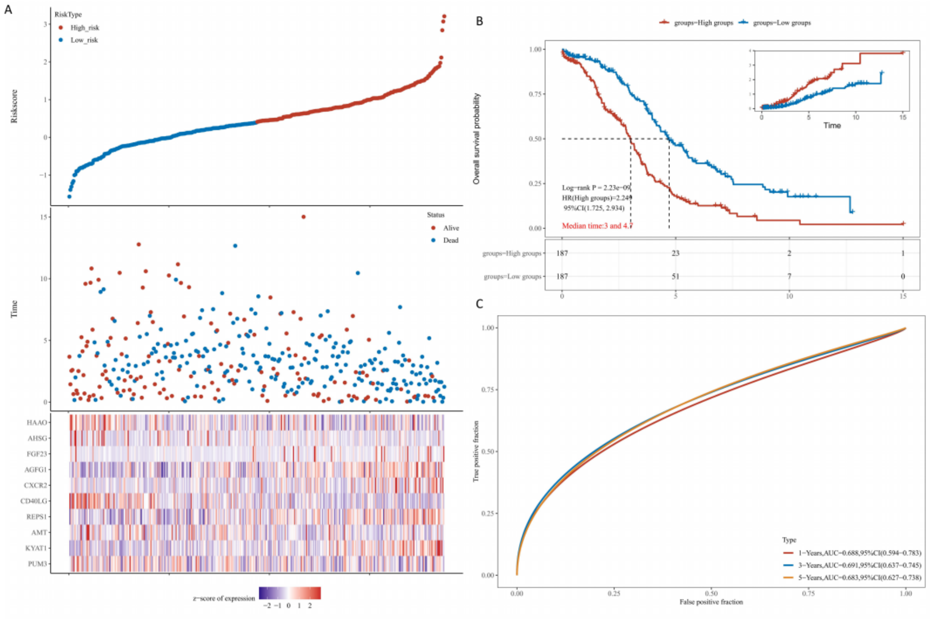

Fig. S1 The validation of 11 CRGs using the TCGA database is crucial for OV. A The gene expression of PUM3, KYAT1, AMT, REPS1, CD40LG, CXCR2, AGFG1, FGF23, AHSG, and HAAO, survival time and survival status of TCGA dataset. The top scatterplot represents the gene expression from low to high. Different colors represent different groups. The scatter plot distribution represents the gene expression of different samples correspond to the survival time and survival status. The bottom figure is the gene expression heatmap; **B** Kaplan-Meier survival analysis of PUM3, KYAT1, AMT, REPS1, CD40LG, CXCR2, AGFG1, FGF23, AHSG, and HAAO from TCGA dataset, comparison among different groups was made by log-rank test. HR (High exp) represents the hazard ratio of the low-expression sample relatives to the high-expression sample. HR> 1 indicates the gene is a risk factor, and HR<1 indicates the gene is a protective factor. HR (95%Cl), the median survival time (LT50) for different groups; **C** The ROC curve and AUC of PUM3, KYAT1, AMT, REPS1, CD40LG, CXCR2, AGFG1, FGF23, AHSG, and HAAO. The higher values of AUC corresponding to higher predictive power.
